# Sustainable partnerships for global nursing education: a Canadian and Brazilian collaboration

**DOI:** 10.1590/1518-8345.0000.3258

**Published:** 2020-05-11

**Authors:** Solina Richter, Greta Cummings, Andrea Bernardes

**Affiliations:** 1 University of Alberta, Faculty of Nursing, Edmonton, AB, Canada.; 2 Universidade de São Paulo, Escola de Enfermagem de Ribeirão Preto, PAHO/WHO Collaborating Centre for Nursing Research Development, Ribeirão Preto, SP, Brazil.

**Figure:**
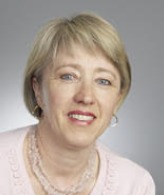


**Figure:**
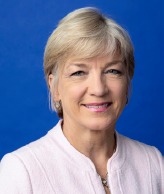


**Figure:**
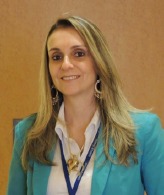


## Introduction

The United Nations Sustainable Development Goals focus on leaving nobody behind and argue that universal health coverage cannot be achieved without strengthening nursing globally^(^ 1 ^)^. It poses several challenges to both nursing education and research to achieve this goal within the increasing complexities in the nursing profession. Nursing faculties have reiterated the need to engage with international partners to combine resources and expertise on an international level to address complex interconnected global nursing and health issues. This commentary will reflect on a sustainable partnership for global nursing education between a Canadian and Brazilian university.

## The need for partnerships

Global collaboration and partnerships have always been a fundamental part of scholarly development. It accelerates the scope, rate, and importance of global research and facilitates our contribution to the global collection of knowledge and expertise on nursing and health-related issues. Combining education and research resources and expertise on an international scale supports the development solutions for complex and interconnected global nursing and health issues. Global collaboration is a prerequisite to bring different perspectives to dialogue on key national and global issues. Nursing education and research partnerships have the potential to strengthen and contribute better health, greater gender equality, and stronger economies; the triple impact of nursing^(^ 2 ^)^. The overarching goal of nursing partnerships is to foster cohesive relationships that support capacity building and strengthening of health systems. The broader context of collaboration relies on the need to partner and collaborate in both nursing education and nursing research. Global collaborations by nursing institutions support the development of faculties that are well equipped to address global health challenges and to understand the complexities of a globalized world^(^ 3 ^)^.

Challenges often exist with establishing international partnerships, although the benefits of these partnerships are well-validated^(^ 4 ^)^. The key component for a successful partnership is the focus on mutual benefits for both partners^(^ 4 ^)^, especially where the partnership is between lower-middle-income and high-income countries. Additionally, another component it the creation of a platform for the exchange of information, skills, and ideas that culminate in improvement in health outcomes^(^ 5 ^)^. Sustainable partnerships include mutual setting of targets, slow, incremental, frequent, and deliberate interaction that regularly reviews progress, regular meetings with succession plans, and strong and committed leadership to monitor mutual goals and strategic contributions and to use resources effectively^(^ 6 ^)^.

## Case example

An example of a successful partnership is the case study of the long-term engagement between the Faculty of Nursing, University of Alberta (FON, UofA) and Ribeirão Preto College of Nursing, University of São Paulo (EERP-USP). The FON at the UofA has developed a strong relationship with EERP-USP. The relationship was initiated in 2004 with a generous donation from a private donor to support a Visiting Scholar Program (VSP). Through this program, Brazilian post-doctoral scholars completed a fellowship at the FON, UofA. The overall goal of the VSP was to promote capacity building in nursing research and enhance international research collaboration between the two institutions. The FON, UofA has hosted over 20 postdoctoral fellows. The program lasted from 2004 until 2017. The UofA FON has also received government-funded students at all levels from Brazil. In return, FON, UofA members visited EERP-USP on short term visits. The collaboration resulted in the development of multiple collaborative research papers and programs highlighting the results of an evaluation with colleagues from both institutions. Research collaboration has developed sustainable collaborative research projects.

Postdoctoral VSP scholars reflected that the experience gave them the opportunity to strengthen their knowledge related to methodological learning processes. The development of teaching skills was not a goal of the VSP, although many scholars reflected on their teaching practices. VSP scholars had the opportunity to attend undergraduate and graduate courses, which contributed to the development of their own teaching practices. Also, FON members visited EERP-USP and participated in research activities, guest lectures, graduate activities, contributing to solidifying the partnership^(^ 7 ^)^. Joint writing of scientific papers for publication between the FON and EERP-USP scholars was experienced as very positive. VSP scholars saw international experiences and connections between international institutions as essential for their career development and to fulfill the mandate of higher education^(^ 8 ^)^.
